# Iron Absorption in Iron-Deficient Women, Who Received 65 mg Fe with an Indonesian Breakfast, Is Much Better from NaFe(III)EDTA than from Fe(II)SO_4_, with an Acceptable Increase of Plasma NTBI. A Randomized Clinical Trial

**DOI:** 10.3390/ph11030085

**Published:** 2018-09-10

**Authors:** Eka Ginanjar, Lilik Indrawati, Iswari Setianingsih, Djumhana Atmakusumah, Alida Harahap, Ina S. Timan, Joannes J. M. Marx

**Affiliations:** 1 Department of Internal Medicine, Faculty of Medicine, University of Indonesia/Dr Cipto Mangunkusumo Hospital, Jakarta 10430, Indonesia; ekginanjar@gmail.com; 2 Department of Clinical Pathology, Faculty of Medicine, University of Indonesia/Dr Cipto Mangunkusumo Hospital, Jakarta 10430, Indonesia; lilikindrawati.dr@gmail.com (L.I.); ina_sutanto@yahoo.com (I.S.T.); 3 Eijkman Institute for Molecular Biology, Jakarta 10430, Indonesia; ning@eijkman.go.id (I.S.); alida@eijkman.go.id (A.H.); 4 Division of Hematology and Medical Oncology, Department of Internal Medicine, Faculty of Medicine, University of Indonesia/Dr Cipto Mangunkusumo Hospital, Jakarta 10430, Indonesia; endjum@hotmail.com; 5 Department of Medical Microbiology, University Medical Centre Utrecht, Heidelberglaan 100, Utrecht 3584 CX, The Netherlands

**Keywords:** iron deficiency anemia, nutrient iron, oral iron therapy, FeSO_4_, NaFeEDTA, non-transferrin-bound iron (NTBI), developing countries, Indonesia

## Abstract

Plasma non-transferrin-bound iron (NTBI) is potentially harmful due to the generation of free radicals that cause tissue damage in vascular and other diseases. Studies in iron-replete and iron-deficient subjects, receiving a single oral test dose of Fe(II)SO_4_ or NaFe(III)EDTA with water, revealed that FeSO_4_ was well absorbed when compared with NaFeEDTA, while only the Fe(II) compound showed a remarkable increase of NTBI. As NaFeEDTA is successfully used for food fortification, a double-blind randomized cross-over trial was conducted in 11 healthy women with uncomplicated iron deficiency. All subjects received a placebo, 6.5 mg FeSO_4_, 65 mg FeSO_4_, 6.5 mg NaFeEDTA, and 65 mg NaFeEDTA with a traditional Indonesian breakfast in one-week intervals. Blood tests were carried out every 60 min for five hours. NTBI detection was performed using the fluorescein-labeled apotransferrin method. Plasma iron values were highly increased after 65 mg NaFeEDTA, twice as high as after FeSO_4_. A similar pattern was seen for NTBI. After 6.5 mg of NaFeEDTA and FeSO_4_, NTBI was hardly detectable. NaFeEDTA was highly effective for the treatment of iron deficiency if given with a meal, inhibiting the formation of nonabsorbable Fe-complexes, while NTBI did not exceed the range of normal values for iron-replete subjects.

## 1. Introduction

Iron deficiency anemia (IDA) is a worldwide health problem, affecting about 2 billion people, particularly in developing countries like Indonesia [1]. IDA has a large impact on productivity, mental performance, child growth, immunity, and pregnancy outcome. About 20 years ago in Indonesia, 25–30% of the population (50 to 70 million subjects) suffered from IDA [2]. The management of iron deficiency by oral iron salts and food iron fortification were effective for raising plasma hemoglobin concentration in Indonesia as demonstrated by large family life surveys on anemia prevalence, estimated in 1997, 2000, and 2008 [3]. The prevalence of anemia decreased in all groups studied, e.g., in women >15 years from 36.0 to 26.6%. Nevertheless, iron deficiency anemia remains a health problem in Indonesia due to insufficient nutrient daily intake [4]. 

Meanwhile, iron supplementation is commonly practiced by physicians in rural and urban areas. Furthermore, the community has easy access to oral iron supplements for their own consumption. Unfortunately, iron drugs are often consumed without a proper diagnosis of iron deficiency anemia, and are even used to combat lethargy or to gain strength and vitality. Measures to prevent iron deficiency should be specifically aimed at population groups at risk because actions to increase iron intake and bioavailability in the general population can be harmful for subjects with undiagnosed homozygous and heterozygous forms of iron overload diseases such as thalassemia intermedia [5].

Despite the fact that plasma-transferrin in iron-deficient patients has a large capacity of free iron-binding sites, treatment with a standard oral dose of 200 mg FeSO_4_ (ferrous sulfate, containing 65 mg of elementary iron) was observed to generate potentially toxic amounts of non-transferrin-bound iron (NTBI) [6]. NTBI is the fraction of iron in plasma that is not tightly and safely bound to transferrin, including a heterogeneous mixture of labile and stable molecular species. NTBI is associated with oxygen radical formation and tissue damage in normal subjects and those with iron overload diseases [7]. In addition, NTBI and many other human iron-containing molecules can be utilized by microorganisms [8]. Therefore, the detection of NTBI after oral administration of regular iron medication, even in subjects with iron deficiency, needs further investigation. As iron-deficient subjects, with mainly free iron-binding sites on circulating transferrin, may be protected against iron-catalyzed reactive oxygen species, it is not clear whether the detection of NTBI after oral iron therapy can be harmful or is just associated with iron absorption physiology.

Not only should ferrous sulfate be investigated, being the standard treatment of iron deficiency, but also the Fe(III) compounds that have been used since 1950 for food iron fortification. In particular, NaFe(III)EDTA (Ferrazone, Akzo Nobel), recommended by the WHO as the preferred iron fortificant for wheat and maize flour, which are staple foods used by the whole population. Layrisse and co-workers described in 1977 that iron absorption from Fe(III)EDTA was about twice as high as that observed from ferrous sulfate [9].

NaFeEDTA is widely used in Indonesia, not only as a food fortificant, but also for the treatment of iron deficiency in children, which is recommended by the Pediatrician Association of Indonesia (IDAI). It is available as a syrup, Ferrostrane^®^ (Teofarma S.r.l., Pavia, Italy), with one teaspoon (5 mL) containing 34 mg of iron. For adults, three to six teaspoons per day are recommended (102–204 mg of iron).

In previous studies that investigated the iron absorption and generation of NTBI in subjects receiving a single oral therapeutic dose of Fe(II)SO_4_ or NaFe(III)EDTA with water, the absorption of iron from FeSO_4_ was much better when compared with NaFeEDTA, while only the Fe(II) compound showed a remarkable increase of NTBI. This was found in iron-replete [10] and in iron-deficient subjects [11].

In the present clinical crossover study in iron-deficient women (without signs or symptoms of inflammation), the post-absorption values for plasma iron and NTBI were measured during five hours from both a therapeutic elementary iron dose (65 mg) and a dose relevant for iron fortification (6.5 mg) in the form of Fe(II)SO_4_ and NaFe(III)EDTA, all presented after a traditional Indonesian breakfast.

## 2. Results

### 2.1. Selection of Participants and Data Collection

After screening 63 apparently healthy female candidates, 11 were selected for a randomized clinical trial as described in the Materials and Methods. All selected subjects fulfilled the criteria for iron deficiency anemia with low values for hemoglobin (Hb), mean cellular volume (MCV), mean cellular hemoglobin (MCH), low seum iron (SI), and high total iron binding capacity (TIBC). Mean transferrin saturation was 7.7% and serum ferritin was less than 20 µg/L These basic data were obtained from examination on minute 0 on the first day of the study when all subjects were only given the test meal: Group A received FeSO_4_ first, and Group B received NaFeEDTA first. There was no significant difference between any of the laboratory characteristics of the subjects from both groups. 

In some samples, taken before the consumption of the standard meal, a raised level of C-reactive protein (CRP) (>10 mg/L) was found, which was interpreted as a sign of inflammation. As iron absorption is decreased during inflammation [12], such episodes were removed from the final results. We also decided to remove investigation episodes if hemolytic blood samples were identified in the laboratory. The minimum number of episodes that could be analyzed, however, was never below six. In all three subjects with increased CRP, of whom the results were removed from the study, serum iron and NTBI curves after iron ingestion were flat, even after the administration of 65 mg Fe as NaFeEDTA, and were no different from the 0 mg Fe placebo dose.

The complete set of data collected during this investigation is available in the Appendix. One table indicates which data were excluded from further evaluation due to hemolysis of the blood sample or CRP-values > 10 mg/L as a nonspecific sign of inflammation. If a test subject’s data for FeSO_4_ had to be removed, data for NaFeEDTA for the same iron dose (6.5 or 65 mg) were also removed.

### 2.2. Results for Iron Absorption and NTBI Generation

**Figure 1:**
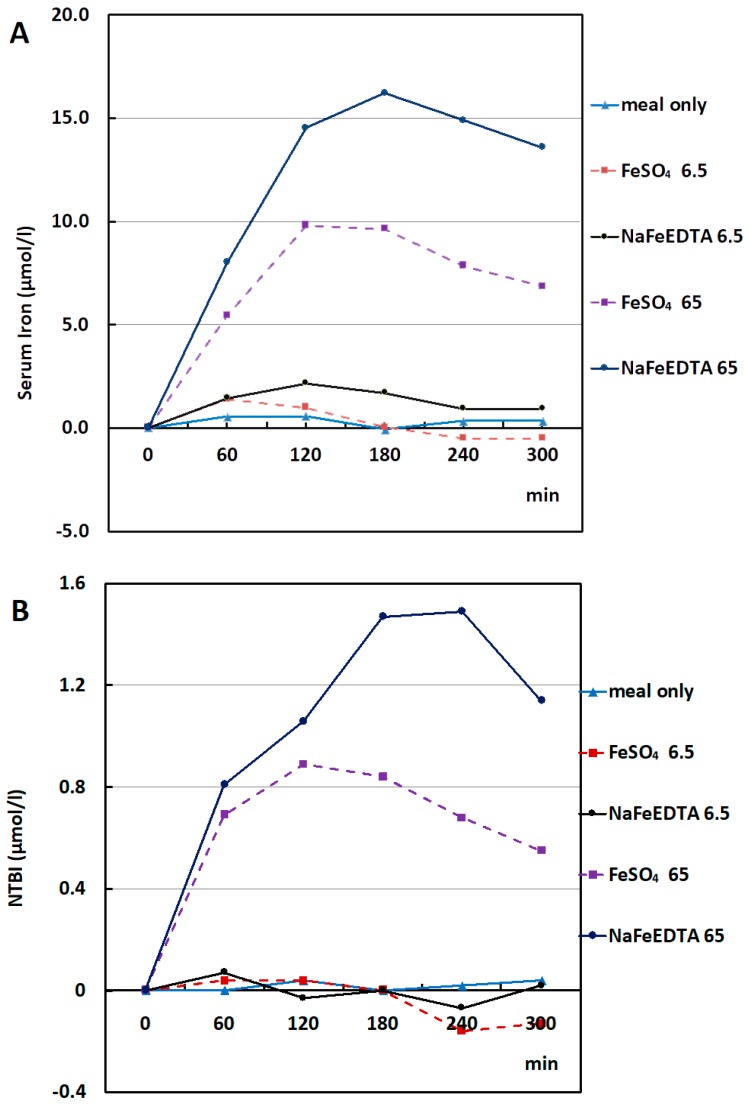
Increase of serum iron (**A**) and serum NTBI (**B**) after 6.5 and 65 mg iron administered after a standard meal as Fe(II)SO_4_ or as NaFe(III)EDTA.

Presented are the results of the increase of serum iron values (Figure 1A) and NTBI (Figure 1B) after donation of one meal only and a meal with the addition of 6.5 or 65 mg iron as either FeSO_4_ or NaFeEDTA. Such simple values represent the dynamic processes after ingestion of the meal with the specified test dose during migration through the stomach and upper intestine (all with a distinct function in iron absorption), and the binding of iron to and the release from transferrin in plasma.

Accumulatively, the increase of serum iron and NTBI after the different oral doses of FeSO_4_ and NaFeEDTA is demonstrated in Table 1 with the area under the curve (AUC) according to Conway et al. [13]. From the calculation of AUC, NTBI generation after 65 mg FeSO_4_ and 65 mg NaFeEDTA was far higher when compared to the placebo and iron supplement of a lower dosage. NTBI generated after 65 mg NaFeEDTA was higher when compared to 65 mg FeSO_4_.

With the method used, hardly any increase in serum iron and NTBI was observed after a meal only (placebo 0 mg Fe), or 6.5 mg iron as either FeSO_4_ or NaFeEDTA. Additionally, no significant difference could be seen for both parameters for all time points after the donation of 6.5 mg iron between FeSO_4_ and NaFeEDTA.

There was a significant difference, however, in the increment of both serum iron and NTBI levels (*p* < 0.05) in subjects given a standard therapeutic dose of 65 mg Fe of both FeSO_4_ and NaFeEDTA. It was remarkable, however, that both iron absorption (estimated by an increase of plasma iron) and the appearance of potentially harmful NTBI were about twice as high after the donation of NaFeEDTA than after FeSO4.

In Table 2, more detailed data are presented for the NTBI values after the administration of 0 mg (placebo) and the high, but therapeutically most relevant, dose of 65 mg FeSO_4_ and NaFeEDTA. In both cases, a considerable increase of NTBI was observed.

### 2.3. Complications and Side Effects

Blood loss. Due to this study protocol, all participants had considerable blood loss. During each of the five test episodes, there was 50 mL blood loss (10 mL at minute 0 min, then 5 times 8 mL) with a total of 250 mL. During the test period, hemoglobin concentration decreased around 0.7 g/dL. Therefore, at the end of the study protocol, all participants received iron supplement therapy. 

Phlebitis in subject 11 after phlebotomy at the administration of 6.5 mg NaFeEDTA. Topical heparin (Thrombophob^®^) was applied and resolved the problem after three days.

Nausea in subject 1 after the administration of 6.5 mg NaFeEDTA, in subject 4 after the administration of 65 mg NaFeEDTA and in subject 10 after the administration of 6.5 mg FeSO_4_. All three subjects were given one omeprazole 20 mg capsule and one domperidone 10 mg tablet. The symptoms were resolved after 30 min.

Other side effects or complications were not observed.

## 3. Discussion

### 3.1. Comparison with Results from Studies Using Similar and Different Methodologies

Numerous clinical studies on the absorption of iron for the treatment or prevention of iron deficiency have been published [14,15]. The method used in the present investigation is not able to provide absolute quantitative values, but iron uptake from the gut after presenting different compounds and amounts of iron can be compared. However, there is a limitation as small increases of non-radiolabelled iron in plasma are undetectable if low iron doses are given.

In this study, iron uptake and the appearance of potentially harmful non-transferrin-bound iron (NTBI) were investigated by administering doses relevant for iron therapy (65 mg Fe) and also for food iron fortification (6.5 mg) to healthy, iron-deficient Indonesian females after an Indonesian standard breakfast. The compounds were Fe(II)SO_4_ (medication of choice for treatment of iron deficiency anemia) and NaFe(III)EDTA (supplement of choice for the fortification of food products and therefore for the prevention of iron deficiency anemia). Intestinal iron uptake was estimated by comparing the serum iron concentration during six hours, and increase of serum NTBI [6]. We compared our results with investigations from Guatemala, published in 2012 [10] and 2013 [11], to try to answer similar questions and use the same experimental approach, however, with a completely different outcome. In all three studies, NTBI tests were performed in the same laboratory in Utrecht.

In the 2012 study [10], an oral supplement of a 0 mg Fe placebo dose in 200 mL water, a therapeutic dose of 100 mg iron as FeSO_4_ (as a commercial syrup dissolved in 200 mL water) or 100 mg of iron as 770 mg of NaFeEDTA powder dissolved in 200 mL of water, was administered to healthy, iron-replete males after at least eight hours of fasting. In this cross-over study, each subject served as his own control. Venepuncture was performed at 0, 90, 180, and 270 min. In the 2013 study [11], the design was identical, in particular, the donation of iron with water, but the test subjects were non-pregnant healthy women with low ferritin values (<30 ng/mL). After 100 mg iron as FeSO_4_, a considerable increase of serum iron was seen in men with adequate iron stores and normal CRP, and a much higher increase in females with low iron stores and normal CRP. Iron uptake from 100 mg iron as NaFeEDTA in men was identical to that from water. Additionally, in iron-deficient females, the serum iron values were only a bit higher.

Troesch et al. [16] and Brittenham et al. [17] used stable isotopes to estimate the percentages of iron absorption in post-absorption curves, and iron utilization by circulating erythrocytes 14 days after ingestion of the labeled test doses. Troesch et al. investigated iron uptake from 6 mg iron as FeSO_4_ with ascorbic acid (AA) and from 6 mg iron as NaFeEDTA. Hardly any increase was seen for both FeSO_4_ and NaFeEDTA when the serum iron values were measured. When labeled with stable isotopes, the 6 mg iron uptake curves were almost identical with those found by us after the uptake of 65 mg iron. After 6 mg iron, the increase of serum iron was higher after FeSO_4_ with AA than after NaFeEDTA.

Brittenham studied women with replete and reduced iron stores, receiving a 6 mg or a 60 mg iron dose as FeSO_4_ with a standard meal. Red blood cell iron utilization was measured using stable isotopes. With this approach, the values for real iron absorption could be calculated, which were higher for iron-deficient than for iron-replete women. Of interest was the percentage of iron absorption in iron-deficient women from the test dose given with a meal, which was 1.22 mg (20.4%) from the 6 mg dose, and 8.82 mg (10.0%) from the 60 mg dose. Peak values for NTBI were 0.1 µmol/L after the 6 mg dose and 0.81 µmol/L after 60 mg Fe. These values were almost identical with those from the comparable doses in our study. After the donation of 65 mg Fe as NaFeEDTA, the mean peak NTBI values were twice as high, but remained below 1.5 µmol/L.

Although the design of our study was similar to the two from Guatemala, there were crucial differences. Most importantly, our iron test dose was not given after eight hours of fasting but directly after ingestion of a standard breakfast. This resulted in a high uptake from 65 mg iron as NaFeEDTA, even twice as high as that from 65 mg Fe as FeSO_4_ (Figure 1A). The conclusion was that the absorption of iron was much better if given with a meal than with water. The pattern for NTBI in plasma was almost identical, suggesting that NaFeEDTA may be less safe than FeSO_4_ (Figure 1B). In addition to a therapeutic dose of 65 mg Fe, we studied a fortification dose of 6.5 mg Fe. No NTBI could be detected in the serum for both iron compounds. Serum iron values were marginally higher for NaFeEDTA. The absolute NTBI values were low because all subjects were iron deficient. 

### 3.2. Aspects of Iron Absorption

**Figure 2:**
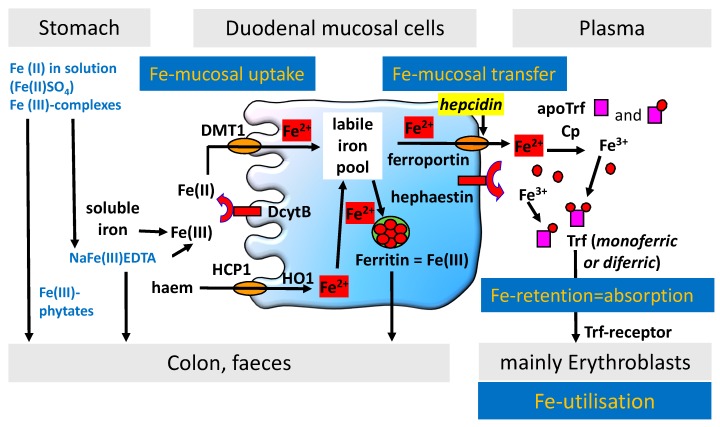
Processing of absorbable iron by the duodenal mucosal cells. DMT1 = divalent metal transporter 1; DcytB = duodenal cytochrome B; HCP1 = hem carrier protein 1; HO1 = hem oxygenase-1; Cp = ceruloplasmin; Trf = transferrin.

If iron is administered in an aqueous solution on an empty stomach, passage through the stomach and upper intestinal tract is rapid. This is an advantage for the uptake of iron from ferrous sulfate as Fe(II) is directly available for transport by the divalent metal transporter (DMT1) to the cytosol of duodenal mucosal cells by simple diffusion [18]. Complexed Fe(III) like in NaFeEDTA must first be reduced to Fe(II) by the reductase duodenal cytochrome B (DcytB) in direct proximity of DMT1. Fe(II) leaves the mucosal cells by ferroportin, mainly depending on the free iron binding sites for Fe(III) on transferrin. Oxidation of Fe(II) by hephestin (membrane) or ceruloplasmin (plasma) is needed for binding to transferrin. The mucosal cell function in processing absorbable iron is summarized in Figure 2.

Plasma iron concentration is the resultant of iron transported to the plasma, free iron-binding sites on transferrin, and iron demand for erythropoiesis, which is increased in iron deficiency and in hemolytic anemias. 

After the duodenum, the nonabsorbed Fe(II) is oxidized in the intestinal lumen and forms complexes with a variety of ligands in more distal parts of the intestinal tract before being removed with the feces. While the results for FeSO_4_ in the Guatemalan studies can be explained by normal physiology, this was not the case with NaFeEDTA administered with water.

EDTA is a chelator that can combine with virtually every metal, depending on its stability constant with the metal. This is influenced by pH, the molar ratio of the chelator to the metal ion, and the presence of competing metal ions and other ligands. Ferric iron has the highest stability constant with EDTA at 25.1 (optimum pH = 1), followed by ferrous iron (14.6) (optimum pH = 5) [19]. The intraluminal pH is rapidly changed from acidic in the stomach (empty state pH 4–5, after meal pH 1.3) to about pH 6 in the duodenum. The pH gradually changes in the small intestine from pH 6 to about pH 7.4 in the terminal ileum, 5.7 in the cecum, and pH 6.7 in the rectum [20].

The binding of EDTA to iron is favored by the acidic environment of the stomach (due to the very high binding constant), but in the more alkaline surroundings of the duodenum, the iron is exchanged in part for other metals. EDTA protects iron in the stomach from inhibitory dietary ligands such as phytates and polyphenols and releases iron in the duodenum, where iron can be absorbed after reduction by DcytB and transport by DMT1. Phytates, present in many cereals and legumes, are powerful inhibitors of iron absorption. Some direct evidence of the ability of NaFeEDTA to prevent their action was obtained in an experiment where bran, a rich source of phytates, was shown to reduce the absorption of iron from ferrous sulfate eleven-fold. In contrast, no such inhibition occurred when bran was fed with NaFeEDTA [21].

Fe(III) reaches the cecum in complexed forms. With EDTA, however, it will form a stable complex. Nonabsorbed Fe as NaFeEDTA may reach the colon with a pH of 5.6 and a relatively low stability constant for NaFe(II)EDTA. Iron absorption from the colon is possible [22]. Some DcytB has been found to be expressed in the large intestine, while ferroportin and DMT1 are expressed at significant levels and are increased in iron deficiency [23]. 

### 3.3. Toxic Effects of NTBI in Plasma after Absorption of Iron

NTBI includes plasma iron that is not bound to transferrin or is a structural part of other proteins in plasma including ferritin. While transferrin-bound iron is redox-inactive and safe, many forms of NTBI are labile and able to exchange iron with other molecular species that can cause the formation of toxic oxygen species and tissue damage. NTBI was first identified in 1978 in patients with beta thalassemia major and intermedia [24]. For decades, NTBI was exclusively associated with (severe) iron overload, although it was also associated with other pathological conditions.

After a publication on the appearance of NTBI in plasma after oral treatment with FeSO_4_ [6] and in hereditary hemochromatosis (HH) heterozygotes [25], the general view was that in these conditions, NTBI might also generate oxygen radicals. In a large epidemiological investigation, high mortality due to myocardial infarction and stroke was detected in female HH heterozygotes, but only in those reported with smoking and/or hypertension as combined risk factors [26]. In another large study investigating the relation of NTBI, serum iron, transferrin saturation, and serum ferritin with the risk of coronary heart disease (CHD) and acute myocardial infarction (AMI), the results did not show an excess risk of CHD or AMI within the highest NTBI tertile when compared with the lowest, but rather seemed to demonstrate a decreased risk [27]. The NTBI was measured with the same method as in the present study, and the total range of NTBI concentrations was −2.06 to 3.51 µmol/L.

As we wanted to compare the post-absorption curves of NTBI in this study, for all compounds and Fe-doses, the 0-min NTBI was set to 0 with the appropriate correction of all NTBI values. When reviewing the complete range of all time-points including the high dose of 65 mg Fe as FeSO_4_ and NaFeEDTA, the range of all values of NTBI was between −0.59 and 0.37 µmol/L. This was very low, and in agreement with the iron-deficient state of the test subjects.

As can be seen in Figure 1, only about 10% of the absorbed iron was identified as NTBI. Many molecular species in plasma can bind iron, however, with a much lower affinity than apotransferrin. This was described in a review by Hider [28]. Most important are citrate and albumin. The citrate level in plasma is 100–120 µmol/L, representing a considerable iron binding capacity. In our test subjects, the total iron binding capacity (TIBC) of transferrin was 45–73 µmol/L. Citrate forms a wide range of oligomeric iron(III) species, which are stable complexes. Transfer of iron between an iron(III) citrate complex and desferrioxamine takes several hours to complete at pH 7.4 [29]. The rate-limiting step is the dissociation of iron from the polynuclear complex. Another molecule able to bind large amounts of iron in plasma is albumin [28]. Its concentration is 34–50 g/L plasma, and albumin possesses a large number of negative carboxylate sites on its surface that are able to bind Fe(III). Absorbed iron bound to citrate or albumin cannot be considered as toxic labile NTBI.

Our conclusion is that humans, after being exposed for a longer time to NTBI values in plasma that can be reached after treatment with highly absorbable iron compounds with a rather moderate increase of serum NTBI, remained healthy. These considerations may contribute to the discussion on the maximum acceptable daily intake of EDTA for iron fortification and iron therapy [30].

### 3.4. Iron Therapy in Developing Countries

Iron is needed for microbial proliferation [8]. There is enormous diversity in the mechanisms of iron uptake and of iron species that can be processed. A minority of pathogenic microorganisms are able to use plasma NTBI, one important species being malaria parasites. This is a huge health problem as malaria, together with iron deficiency anemia, is endemic in many regions of the world [31]. 

Thalassemia has a high prevalence along the Mediterranean coast, Africa, Middle East, India, Burma, Southeast Asia including Indonesia, and Melanesia up to the Pacific Islands [32,33]. A total of 3–10% of the world’s population are β thalassemia carriers with the prevalence in Indonesia reaching 8% [34]. Thalassemia with iron overload and iron deficiency co-exists in the same population. Both are microcytic anemias, and clinical symptoms of iron deficiency and thalassemia heterozygotes are similar. Thalassemia patients frequently receive oral iron supplements for six months for up to two years. This practice is based on screening difficulties between iron deficiency anemia and anemia in Thalassemia trait or intermedia. Apart from that, the public has access to hematinics that are sold freely over the counter.

The result of this study, showing an increment of NTBI generation after the administration of oral iron supplements may create awareness in healthcare providers to include sufficiently informative laboratory tests before providing oral iron supplements for iron deficiency only and to exclude iron-replete subjects and thalassemia heterozygotes. 

## 4. Materials and Methods

This investigation was a randomized double-blind phase III clinical trial. After randomization, a cross-over design was used. The trial was carried out at the Eijkman Institute Jakarta from November 2008 to April 2009. Laboratory tests were performed at the University of Indonesia/Dr Cipto Mangunkusumo Hospital, Jakarta, Indonesia. NTBI values were measured in the laboratory of the Department of Medical Microbiology, University Medical Centre Utrecht, Utrecht, The Netherlands.

### 4.1. Selection of Test Subjects

**Inclusion criteria:** (1) Female; (2) Age 15–60 years old; (3) Iron deficiency (serum Ferritin < 20 µg/mL); and (4) Informed consent to be involved in the research.

**Exclusion criteria:** (1) Suffering from a chronic illness; (2) Currently having an acute or severe chronic infection; (3) Currently under drug therapy or receiving an iron supplement in any form, minimum one week prior to the clinical investigation; (4) Oral iron supplement allergy; (5) Suffering from a disease or disorder of the alimentary tract; and (6) Pregnancy.

Eleven females were selected after screening 63 apparently healthy women: (1) students of the Faculty of Medicine University of Indonesia who went for Thalassemia screening at the Eijkman Institute for Molecular Biology of Jakarta who were found to have microcytic hypochromic anemia; and (2) healthy women from several areas in Jakarta who were known to be iron deficiency patients at the Hematology and Medical Oncology outpatient clinic in the department of Internal Medicine RSCM. Eleven iron-deficient females were finally selected for this investigation.

The age range of the research subjects was between 15 and 34 years with a mean age of 24.18 years. A major portion of the study subjects were of a low education level (seven persons) and nine persons of low income, thus can be assumed to be of low socioeconomic level. A description of the selected test subjects is provided in Table 3. 

### 4.2. Procedure of the Investigation

The selected subjects were asked to visit the Eijkman Institute five times with one-week intervals to undergo the test procedure.

**Scheme 1:**
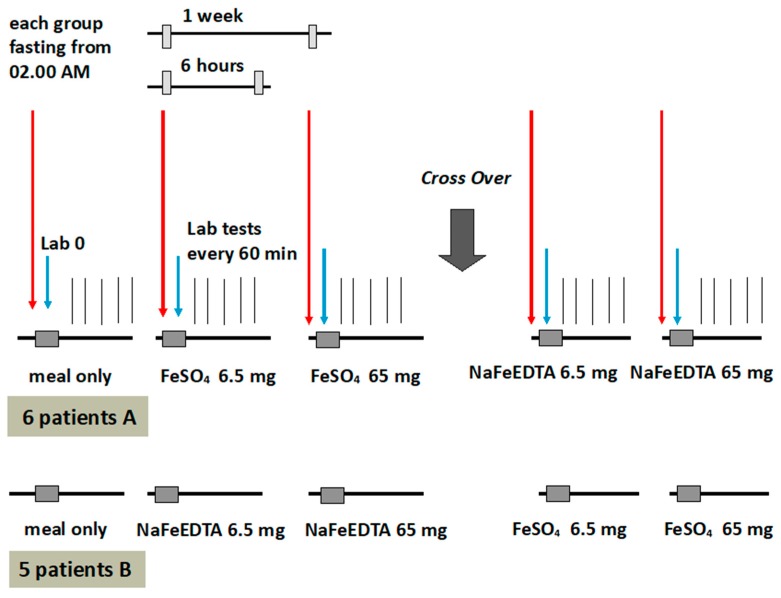
Iron supplement administration schedule and iron doses.

The subjects were divided into two groups of six (A) and five (B) subjects. A cross-over procedure was used as shown in Scheme 1. The procedure sequence towards the subjects was determined by double-blind randomization using a voting technique. Randomization and crossing-over were performed to determine who would receive either FeSO_4_ or NaFeEDTA first. Each subject needed to attend five times with a one-week interval as a wash-out period.

Before any test episode (with or without receiving an iron dose), the test subjects were not allowed to take anything orally except plain water for at least six hours prior to the study. An intravenous catheter (Veinplon^®^) with a saline lock method was applied to enable multiple blood drawings in all subjects. A quantity of 10 mL blood was taken for the first venous blood drawing for Hb, MCV, MCH, MCHC, NTBI, Ferritin, SI, TIBC, transferrin saturation, CRP, and hepcidin. 

**Scheme 2:**
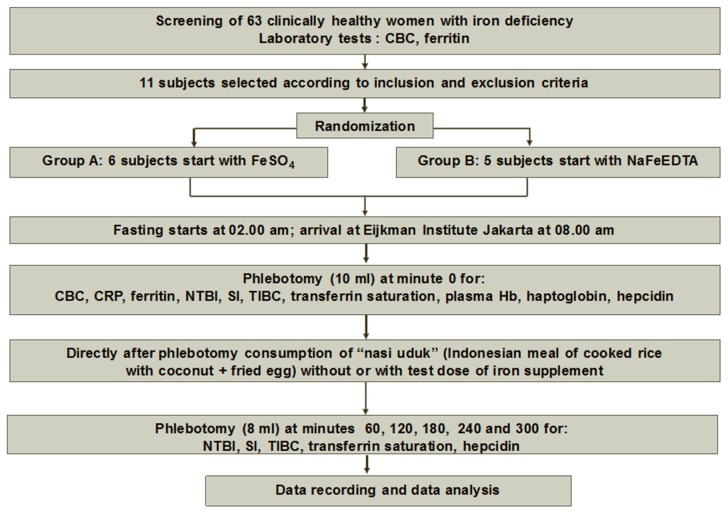
Algorithm of the investigation.

Next, subjects were asked to consume a meal of rice with coconut milk (*nasi uduk*) and omelet, which is low in iron content, followed by the oral iron supplementation as shown in the table (see Scheme 2).

The mean reason why a meal prior to supplementation was included in the protocol was the reduction of side effects of oral iron supplementation like nausea, vomiting, epigastric pain, and burning sensation in the chest. A placebo dose was included to see the influence of the standard meal, and eventually circadian fluctuations of serum iron and NTBI.

After each test dose of oral iron supplement, 8 mL venous blood was taken at 60th, 120th, 180th, 240th, and 300th min for the measurement of NTBI, SI, TIBC, and transferrin saturation. Subjects were allowed to take anything orally after one hour of the test dose of oral iron supplement. Low iron snacks were provided during the whole procedure because subjects were at the research area for about six hours.

### 4.3. Production of NaFeEDTA Capsules

Ferric sodium EDTA (C_10_H_12_FeN_2_NaO_8_3H_2_O) MW = 421.1 was provided by Akzo Nobel, The Netherlands, as Ferrazone^®^. The capsules were produced in the Pharmacy of Dr. Cipto Mangunkusumo Hospital, Jakarta, Indonesia. One capsule with 65 mg elementary iron was composed of 490 mg NaFeEDTA, and 110 mg lactose. One capsule with 6.5 mg elementary iron consisted of 49 mg NaFeEDTA and 600 mg lactose.

### 4.4. Composition of Test Meal

Before taking the test dose of the iron supplement, all subjects consumed “*nasi uduk*” (a standard Indonesian meal of cooked rice with coconut + fried egg). This meal is free from fortified iron and has a very low amount of natural iron. Composition of one portion of rice 100 g = 178 calories: protein: 6.8 g; fat: 0.7 g; carbohydrate: 78.9 g; and iron: 0.5 mg. One portion of fried egg (50 g) = 105.8 calories: protein: 9.3 g; fat: 14.6 g; carbohydrate: 1.5 g; and iron: 2.7 mg.

### 4.5. Laboratory Test Methods

Routine laboratory tests were performed by the Laboratory of Clinical Pathology, Faculty of Medicine, University of Indonesia/Dr Cipto Mangunkusumo Hospital. 

Laboratory test methods included hemoglobin: cyanide-free sodium lauryl sulfate (SLS) Sysmex XT-2000i; erythrocytes, leukocytes, hematocrit, MCV, MCH, MCHC: flow cytometry with semiconductor laser hydrodynamic focusing (Sysmex XT-2000i); serum iron and total iron binding capacity: Ferrozine (Cobas Integra 400, Roche Diagnostics, Risch-Rotkreuz, Switzerland) Transferrin saturation %: SI/TIBC x %; C-reactive protein (CRP): turbidimetric (Cobas Integra 400)

Non-transferrin iron (NTBI): fluorescein-labeled apotransferrin (Fl-aTf) [35] was performed at the Eijkman-Winkler Institute for Microbiology, Infectious Diseases, and Inflammation, University Medical Centre Utrecht, The Netherlands. Frozen samples were sent from Jakarta to Utrecht.

### 4.6. Estimation of Iron Absorption

For the estimation of iron absorption from the gut, the most superior technique is a double isotope method where iron is labeled with Fe^59^ and a nonabsorbable substance (^51^CrCl), while the accumulation of both radioactive isotopes in the body can be measured simultaneously with a whole-body counter (WBC) [36]. As such methods, using in vivo radioactive material and very expensive equipment, are no longer available, stable isotopes or chemical laboratory techniques are used for comparing iron uptake from two different compounds. For a comparison of iron absorption from two non-labeled different iron compounds, with or without a meal, in different iron-doses, either Fe(II) or Fe(III), a comparison of serum iron curves is considered a reliable and powerful tool [13,37]. Absolute values for total iron absorption, however, cannot be estimated with this method.

### 4.7. Data Management and Analysis

Research data were recorded in tested research questionnaires. Verified data were analyzed and organized in text format, tables, or figures using SPSS version 16.0 and NCSS 2007 research software. Multivariate analysis was carried out using one-way ANOVA parametric tests to see the difference in NTBI level generation in each test dose of iron supplement and placebo every hour. Normality tests were performed before using the Shapiro–Wilk method in view of the small number of subjects (11 persons with *n* < 50). The result of the normality tests showed a slightly abnormal distribution of data by which log10 transformation was carried out. However, in 6.5 mg FeSO_4_ from the 240th min, the distribution was still abnormal, thus a nonparametric Kruskal–Wallis test was performed followed by a Mann–Whitney test to have a look at the significance between test doses in the 240th min. One-way ANOVA was performed followed by post hoc analysis.

### 4.8. Ethical Assessment

Signed, informed consent was obtained from all test subjects before participation in the study. The study was conducted in accordance with the Declaration of Helsinki. Ethical assessment of the study protocol was conducted by the Ethical Committee of the Faculty of Medicine, University of Indonesia/Dr Cipto Mangunkusumo Hospital and was approved on 13 October 2008. The Project Identification Number is 338/PT02.FK/ETIK/2008.

The original protocol also contained an investigation, similar to the iron-deficient subjects, in apparently healthy females with nontransfused alpha thalassemia intermedia. This part of the study was not approved as it implicated the donation of iron to a potentially endangered group of patients.

## 5. Conclusions

1. In healthy iron-deficient females, intestinal iron uptake from NaFe(III)EDTA, provided as a therapeutic dose of 65 mg Fe, was twice as high as that from Fe(II)SO_4_ if ingested with a traditional Indonesian meal.
2. Measured in the same samples, non-transferrin-bound iron (NTBI) also increased with an almost identical time-related pattern.
3. When a 6.5 mg iron dose (representing iron fortification) was ingested, the post-absorption curves and increase of NTBI of NaFeEDTA and FeSO_4_ were almost flat, being below the detection level of the method used. Such studies should be performed with stable isotopes, estimating red blood cell iron utilization.
4. For NTBI, the post-absorption concentration in plasma was representative for the whole circulation being the dilution volume. If the NTBI values remain within the range considered to be normal for iron-replete men after the therapeutic dose of 65 mg iron, then one should not fear pathological side effects.
5. Although iron absorption from NaFeEDTA was almost zero if given with water, as was demonstrated for iron-replete men and women, its absorption was excellent (and even much better than that from FeSO_4_), if given with a meal as described in this study.

## Figures and Tables

**Table 1:** Area under the curve (AUC) of serum iron and NTBI during 300 min after ingestion.

Test Dose	AUC Serum Iron (µmol/L)	AUC NTBI (µmol/L)
Fe 0.0 mg (placebo)	100	3.0
6.5 mg FeSO_4_	89	−10.5
65 mg FeSO_4_	2017	203.7
6.5 mg NaFeEDTA	370	−1.8
65 mg NaFeEDTA	2968	324

**Table 2:** NTBI (µmol/L) generation after ingestion of a meal, followed by 0 mg Fe (placebo), 65 mg FeSO_4_ and 65 mg NaFeEDTA.

**Minute**	**Placebo**	**65 mg FeSO_4_**	***p*-Value**
0	0	0	-
60	−0.0034 ± 0.1798	0.6866 ± 0.7498	0.002
120	0.0433 ± 0.1565	0.8886 ± 0.9156	0.012
180	−0.0031 ± 0.1781	0.8412 ± 0.7522	<0.001
240	0.0154 ± 0.2372	0.6795 ± 0.4329	0.001
300	0.0409 ± 0.2194	0.5531 ± 0.6003	0.020
**Minute**	**Placebo**	**65 mg NaFeEDTA**	***p*-Value**
0	0	0	-
60	−0.0034 ± 0.1798	0.8106 ± 0.8073	0.007
120	0.0433 ± 0.1565	1.0566 ± 0.7181	0.002
180	−0.0031 ± 0.1781	1.4695 ± 0.7247	<0.001
240	0.0154 ± 0.2372	1.4922 ± 0.7523	<0.001
300	0.0409 ± 0.2194	1.1421 ± 0.7509	<0.001
**Minute**	**65 mg FeSO_4_**	**65 mg NaFeEDTA**	***p*-Value**
0	0	0	-
60	0.6866 ± 0.7498	0.8106 ± 0.8073	0.530
120	0.8886 ± 0.9156	1.0566 ± 0.7181	0.423
180	0.8412 ± 0.7522	1.4695 ± 0.7247	0.017
240	0.6795 ± 0.4329	1.4922 ± 0.7523	0.001
300	0.5531 ± 0.6003	1.1421 ± 0.7509	0.011

**Table 3:** Distribution characteristics of the selected subjects.

Characteristic	Mean	Reference Value
	(n = 11)	
Age	24.18	
Education		
Low	7 subjects	
Medium	3 subjects	
High	1 subject	
Income		
Low	9 subjects	
Medium	2 subjects	
High	-	
Hemoglobin	10.9	12–14 gr/dL
MCV	74.9	82–92 fl
MCH	24.6	27–31 pg
MCHC	32.9	32–36 g/dL
Serum Iron	4.8	6.6–26.0 µmol/L
TIBC	76.9	44.6–73.4 µmol/L
Transferrin Saturation	7.7	<15% = Iron Deficiency
Ferritin	6.2	20–300 µg/L

## Supplementary Materials

Tables presenting a complete data set of serum iron and NTBI data. Annex 1: Complete set of serum iron values. Annex 2: Complete set of NTBI values.
