# Supplementary material for: Iron Absorption in Iron-Deficient Women, Who Received 65 mg Fe with an Indonesian Breakfast, Is Much Better from NaFe(III)EDTA than from Fe(II)SO4, with an Acceptable Increase of Plasma NTBI. A Randomized Clinical Trial
Source: Pharmaceuticals (Basel). 2018 Sep 10;11(3):85. doi: 10.3390/ph11030085 (PMC6161297; doi:10.3390/ph11030085)
Supplement: Supplementary file 1 [file pharmaceuticals-11-00085-s001.zip › suppl/Annex 1; serum iron values.htm]

|  |  |  |  |  |  |  |  |  |  |  |  |  |  |  |  |  |  |  |  |  |
|  | | | | | | | | | | | | | | | | | | | | |
| Mean Table of SI (Without Exclusion) µg/l | | | | | | | | | | | | | |  | | | | | | |
|  |  | 1 | 2 | 3 | 4 | 5 | 6 | 7 | 8 | 9 | 10 | 11 | Mean |  | | | | | | |
| Placebo | 0 | 49 | 47 | 41 | 98 | 13 | 49 | 21 | 85 | 16 | 28 | 44 | 44,6 |  | | | | | | |
|  | 60 | 16 | 68 | 54 | 43 | 16 | 52 | 16 | 34 | 22 | 26 | 43 | 35,5 |  | | | | | | |
|  | 120 | 19 | 27 | 64 | 41 | 23 | 39 | 13 | 31 | 23 | 25 | 26 | 30,1 |  | | | | | | |
|  | 180 | 15 | 35 | 51 | 30 | 16 | 44 | 12 | 36 | 18 | 25 | 31 | 28,5 |  | | | | | | |
|  | 240 | 22 | 24 | 68 | 34 | 16 | 40 | 19 | 33 | 10 | 26 | 33 | 29,5 |  | | | µ |  | | |
|  | 300 | 15 | 28 | 63 | 35 | 17 | 33 | 20 | 32 | 13 | 33 | 32 | 29,2 |  | | | | | | |
| FeSO4 6,5 | 0 | 79 | 16 | 30 | 42 | 9 | 51 | 16 | 74 | 12 | 23 | 34 | 35,1 |  | | | | | | |
|  | 60 | 68 | 18 | 51 | 32 | 14 | 46 | 12 | 69 | 12 | 20 | 23 | 33,2 |  | | | | | | |
|  | 120 | 54 | 24 | 48 | 29 | 14 | 44 | 12 | 63 | 21 | 14 | 29 | 32,0 |  | | | | | | |
|  | 180 | 41 | 15 | 44 | 26 | 6 | 34 | 14 | 17 | 52 | 10 | 24 | 25,7 |  | | | | | | |
|  | 240 | 31 | 12 | 51 | 27 | 15 | 27 | 10 | 39 | 17 | 9 | 24 | 23,8 |  | | | | | | |
|  | 300 | 25 | 23 | 47 | 22 | 14 | 25 | 17 | 32 | 21 | 16 | 16 | 23,5 |  | | | | | | |
| FeSO4 65 | 0 | 23 | 38 | 26 | 47 | 14 | 26 | 13 | 24 | 18 | 23 | 18 | 24,5 |  | | | | | | |
|  | 60 | 30 | 146 | 55 | 44 | 44 | 82 | 32 | 54 | 34 | 140 | 27 | 62,5 |  | | | | | | |
|  | 120 | 90 | 113 | 111 | 108 | 34 | 74 | 21 | 42 | 26 | 155 | 33 | 73,4 |  | | | | | | |
|  | 180 | 110 | 108 | 101 | 111 | 33 | 68 | 23 | 43 | 26 | 119 | 59 | 72,8 |  | | | | | | |
|  | 240 | 88 | 86 | 69 | 83 | 28 | 66 | 20 | 36 | 21 | 90 | 118 | 64,1 |  | | | | | | |
|  | 300 | 78 | 76 | 55 | 89 | 16 | 66 | 27 | 24 | 18 | 70 | 127 | 58,7 |  | | | | | | |
| NaFeEDTA 6,5 | 0 | 18 | 23 | 13 | 22 | 19 | 19 | 23 | 44 | 18 | 41 | 23 | 23,9 |  | | | | | | |
|  | 60 | 14 | 31 | 29 | 24 | 36 | 22 | 55 | 43 | 24 | 31 | 29 | 30,7 |  | | | | | | |
|  | 120 | 21 | 41 | 23 | 40 | 41 | 22 | 34 | 53 | 26 | 36 | 51 | 35,3 |  | | | | | | |
|  | 180 | 18 | 39 | 26 | 36 | 38 | 20 | 28 | 58 | 27 | 29 | 44 | 33,0 |  | | | | | | |
|  | 240 | 11 | 33 | 26 | 40 | 34 | 13 | 23 | 45 | 19 | 33 | 41 | 28,9 |  | | | | | | |
|  | 300 | 15 | 24 | 26 | 37 | 26 | 13 | 25 | 42 | 15 | 45 | 42 | 28,2 |  | | | | | | |
| NaFeEDTA 65 | 0 | 6 | 16 | 35 | 36 | 15 | 14 | 18 | 42 | 15 | 37 | 23 | 23,4 |  | | | | | | |
|  | 60 | 14 | 27 | 92 | 47 | 123 | 19 | 26 | 55 | 10 | 122 | 124 | 59,9 |  | | | | | | |
|  | 120 | 39 | 107 | 130 | 85 | 151 | 8 | 57 | 91 | 17 | 110 | 188 | 89,4 |  | | | | | | |
|  | 180 | 78 | 156 | 161 | 85 | 143 | 16 | 48 | 100 | 17 | 102 | 170 | 97,8 |  | | | | | | |
|  | 240 | 104 | 179 | 145 | 83 | 97 | 16 | 45 | 108 | 27 | 83 | 132 | 92,6 |  | | | | | | |
|  | 300 | 109 | 185 | 132 | 62 | 82 | 7 | 37 | 99 | 23 | 65 | 139 | 85,5 |  | | | | | | |
|  | Note: |  | Hemolytic Condition | |  |  |  |  |  |  |  |  |  |  | | | | | | |
|  | | | | | | | | | | | | | | | | | | | | |
| Modification Result of SI (With Exclusion); µg/l | | | | | | | | | | | | | | |  | | | | | |
|  | Time | 1 | 2 | 3 | 4 | 5 | 6 | 7 | 8 | 9 | 10 | 11 | Mean | Subject |  | | | | | |
| Placebo | 0 | Hemolysis | 47 | 41 | Hemolysis | 13 | 49 | 21 | Hemolysis | 16 | 28 | Hemolysis | 30,7 | 7 |  | | | | | |
|  | 60 | 16 | Hemolysis | 54 | 43 | 16 | 52 | 16 | 34 | 22 | 26 | 43 | 32,2 | 10 |  | | | | | |
|  | 120 | 19 | 27 | 64 | 41 | 23 | 39 | 13 | 31 | 23 | 25 | 26 | 30,1 | 11 |  | | | | | |
|  | 180 | 15 | 35 | 51 | 30 | 16 | 44 | 12 | 36 | 18 | 25 | 31 | 28,5 | 11 |  | | | | | |
|  | 240 | 22 | 24 | 68 | 34 | 16 | 40 | 19 | 33 | 10 | 26 | 33 | 29,5 | 11 |  | | | | | |
|  | 300 | 15 | 28 | 63 | 35 | 17 | 33 | 20 | 32 | 13 | 33 | 32 | 29,2 | 11 |  | | | | | |
| FeSO4 6,5 | 0 | Hemolysis | 16 | 30 |  | 9 |  | 16 | 74 |  | 23 | 23 | 27,3 | 7 |  | | | | | |
|  | 60 | 68 | 18 | 51 |  | 14 |  | 12 | 69 |  | 20 | 23 | 34,4 | 8 |  | | | | | |
|  | 120 | 54 | 24 | 48 | Skip cause | 14 |  | 12 | 63 | Skip cause | 14 | 29 | 32,3 | 8 |  | | | | | |
|  | 180 | 41 | 15 | 44 | CRP 15.1 | 6 |  | 14 | 17 | CRP 28.5 | 10 | 24 | 21,4 | 8 |  | | | | | |
|  | 240 | 31 | 12 | 51 |  | 15 |  | 10 | 39 | Fer>26.8 | 9 | 24 | 23,9 | 8 |  | | | | | |
|  | 300 | 25 | 23 | 47 |  | 14 |  | 17 | 32 |  | 16 | 16 | 23,8 | 8 |  | | | | | |
| FeSO4 65 | 0 | 23 | 38 | 26 | 47 | 14 |  | 13 | 24 |  | 23 | 18 | 25,1 | 9 |  | | | | | |
|  | 60 | 30 | Hemoysis | 55 | 44 | 44 |  | 32 | 54 |  | 140 | 27 | 53,3 | 8 |  | | | | | |
|  | 120 | 90 | 113 | 111 | 108 | 34 |  | 21 | 42 |  | 155 | 33 | 78,6 | 9 |  | | | | | |
|  | 180 | 110 | 108 | 101 | 111 | 33 |  | 23 | 43 |  | 119 | 59 | 78,6 | 9 |  | | | | | |
|  | 240 | 88 | 86 | 69 | 83 | 28 |  | 20 | 36 |  | 90 | 118 | 68,7 | 9 |  | | | | | |
|  | 300 | 78 | 76 | 55 | 89 | 16 |  | 27 | 24 |  | 70 | 127 | 62,4 | 9 |  | | | | | |
| NaFeEDTA 6,5 | 0 | 18 | 23 | 13 |  | 19 |  | 23 | 44 |  | 41 | 23 | 25,5 | 8 |  | | | | | |
|  | 60 | 14 | 31 | 29 |  | 36 |  | 55 | 43 |  | 31 | 29 | 33,5 | 8 |  | | | | | |
|  | 120 | 21 | 41 | 23 |  | 41 | Skip cause | 34 | 53 |  | 36 | 51 | 37,5 | 8 |  | | | | | |
|  | 180 | 18 | 39 | 26 |  | 38 | CRP 13.3 | 28 | 58 |  | 29 | 44 | 35,0 | 8 |  | | | | | |
|  | 240 | 11 | 33 | 26 |  | 34 |  | 23 | 45 |  | 33 | 41 | 30,8 | 8 |  | | | | | |
|  | 300 | 15 | 24 | 26 |  | 26 |  | 25 | 42 |  | 45 | 42 | 30,6 | 8 |  | | | | | |
| NaFeEDTA 65 | 0 | 6 | 16 | 35 | 36 | 15 |  | 18 | 42 |  | 37 | 23 | 25,3 | 9 |  | | | | | |
|  | 60 | 14 | 27 | 92 | 47 | 123 |  | 26 | 55 |  | 122 | 124 | 70,0 | 9 |  | | | | | |
|  | 120 | 39 | 107 | 130 | 85 | 151 | Skip cause | 57 | 91 | Skip cause | 110 | 188 | 106,4 | 9 |  | | | | | |
|  | 180 | 78 | 156 | 161 | 85 | 143 | CRP 110 | 48 | 100 | CRP 47 | 102 | 170 | 115,9 | 9 |  | | | | | |
|  | 240 | 104 | 179 | 145 | 83 | 97 | Fer > 50% | 45 | 108 | Fer > 50% | 83 | 132 | 108,4 | 9 |  | | | | | |
|  | 300 | 109 | 185 | 132 | 62 | 82 |  | 37 | 99 |  | 65 | 139 | 101,1 | 9 |  | | | | | |
|  |  |  |  |  |  |  |  |  |  |  |  |  |  |  |  | | | | | |
|  | Note : |  | Hemolytic Condition | |  |  |  |  |  |  |  |  |  |  |  | | | | | |
|  |  |  | Drop Out |  |  |  |  |  |  |  |  |  |  |  |  | | | | | |
|  |  |  | | |  |  |  |  |  |  |  |  |  |  |  |  |  |  |  |  |
|  | |  |  |  |  |  |  |  | | | | | | | | | | | | |
|  | | | | | | | | | | | | | | | | | | | | |
|  | |  |  |  |  |  |  |  |  |  |  |  |  | | | | | | | |
| Modification Result of SI (With Exclusion); µg/l; Start from 0 | | | | | | | | | | | | | | |  | | | | | |
|  | Time | 1 | 2 | 3 | 4 | 5 | 6 | 7 | 8 | 9 | 10 | 11 | Mean | Subject |  | | | | | |
| Placebo | 0 | Hemolysis | 0 | 0 | Hemolysis | 0 | 0 | 0 | Hemolysis | 0 | 0 | Hemolysis | 0,0 | 7 |  | | | | | |
|  | 60 | -7 | Hemolysis | 13 | 21 | 3 | 3 | -5 | 10 | 6 | -2 | 20 | 6,2 | 10 |  | | | | | |
|  | 120 | -4 | -20 | 23 | 19 | 10 | -10 | -8 | 7 | 7 | -3 | 3 | 2,2 | 11 |  | | | | | |
|  | 180 | -8 | -12 | 10 | 8 | 3 | -5 | -9 | 12 | 2 | -3 | 8 | 0,5 | 11 |  | | | | | |
|  | 240 | -1 | -23 | 27 | 12 | 3 | -9 | -2 | 9 | -6 | -2 | 10 | 1,6 | 11 |  | | | | | |
|  | 300 | -8 | -19 | 22 | 13 | 4 | -16 | -1 | 8 | -3 | 5 | 9 | 1,3 | 11 |  | | | | | |
| FeSO4 6,5 | 0 | Hemolysis | 0 | 0 |  | 0 |  | 0 | 0 |  | 0 | 0 | 0,0 | 7 |  | | | | | |
|  | 60 | 45 | 2 | 21 |  | 5 |  | -4 | -5 |  | -3 | 0 | 7,6 | 8 |  | | | | | |
|  | 120 | 31 | 8 | 18 | Skip cause | 5 |  | -4 | -11 | Skip cause | -9 | 6 | 5,5 | 8 |  | | | | | |
|  | 180 | 18 | -1 | 14 | CRP 15.1 | -3 |  | -2 | -57 | CRP 28.5 | -13 | 1 | -5,4 | 8 |  | | | | | |
|  | 240 | 8 | -4 | 21 |  | 6 |  | -6 | -35 | Fer>26.8 | -14 | 1 | -2,9 | 8 |  | | | | | |
|  | 300 | 2 | 7 | 17 |  | 5 |  | 1 | -42 |  | -7 | -7 | -3,0 | 8 |  | | | | | |
| FeSO4 65 | 0 | 0 | 0 | 0 | 0 | 0 |  | 0 | 0 |  | 0 | 0 | 0,0 | 9 |  | | | | | |
|  | 60 | 7 | Hemoysis | 29 | -3 | 30 |  | 19 | 30 |  | 117 | 9 | 29,8 | 8 |  | | | | | |
|  | 120 | 67 | 75 | 85 | 61 | 20 |  | 8 | 18 |  | 132 | 15 | 53,4 | 9 |  | | | | | |
|  | 180 | 87 | 70 | 75 | 64 | 19 |  | 10 | 19 |  | 96 | 41 | 53,4 | 9 |  | | | | | |
|  | 240 | 65 | 48 | 43 | 36 | 14 |  | 7 | 12 |  | 67 | 104 | 44,0 | 9 |  | | | | | |
|  | 300 | 55 | 38 | 29 | 42 | 2 |  | 14 | 0 |  | 47 | 109 | 37,3 | 9 |  | | | | | |
| NaFeEDTA 6,5 | 0 | 0 | 0 | 0 |  | 0 |  | 0 | 0 |  | 0 | 0 | 0,0 | 8 |  | | | | | |
|  | 60 | -4 | 8 | 16 |  | 17 |  | 32 | -1 |  | -10 | 6 | 8,0 | 8 |  | | | | | |
|  | 120 | 3 | 18 | 10 |  | 22 | Skip cause | 11 | 9 |  | -5 | 28 | 12,0 | 8 |  | | | | | |
|  | 180 | 0 | 16 | 13 |  | 19 | CRP 13.3 | 5 | 14 |  | -12 | 21 | 9,5 | 8 |  | | | | | |
|  | 240 | -7 | 10 | 13 |  | 15 |  | 0 | 1 |  | -8 | 18 | 5,3 | 8 |  | | | | | |
|  | 300 | -3 | 1 | 13 |  | 7 |  | 2 | -2 |  | 4 | 19 | 5,1 | 8 |  | | | | | |
| NaFeEDTA 65 | 0 | 0 | 0 | 0 | 0 | 0 |  | 0 | 0 |  | 0 | 0 | 0,0 | 9 |  | | | | | |
|  | 60 | 8 | 11 | 57 | 11 | 108 |  | 8 | 13 |  | 85 | 101 | 44,7 | 9 |  | | | | | |
|  | 120 | 33 | 91 | 95 | 49 | 136 | Skip cause | 39 | 49 | Skip cause | 73 | 165 | 81,1 | 9 |  | | | | | |
|  | 180 | 72 | 140 | 126 | 49 | 128 | CRP 110 | 30 | 58 | CRP 47 | 65 | 147 | 90,6 | 9 |  | | | | | |
|  | 240 | 98 | 163 | 110 | 47 | 76 | Fer > 50% | 27 | 66 | Fer > 50% | 46 | 109 | 82,4 | 9 |  | | | | | |
|  | 300 | 103 | 169 | 97 | 26 | 67 |  | 19 | 57 |  | 28 | 116 | 75,8 | 9 |  | | | | | |
|  | | | | | | | | | | | | | | | | | | | | |
|  | Note : |  | Hemolytic Condition |  |  | | | | | | | | | | | | | | | |
|  | |  | Drop Out |  |  | | | | | | | | | | | | | | | |
|  | | | | | | | | | | |  |  |  |  |  | | | | | |
|  |  |  |  |  |  |  |  |  | | | | | | | | | | | | |
|  | | | | | | | | | | | | | | | | | | | | |
|  | | | | |  |  | | | | | | | | | | | | | | |
|  |  |  |  |  |  |  |  |  |  |  |  |  |  |  |  |  |  |  |  |  |
