# Supplementary material for: Iron Absorption in Iron-Deficient Women, Who Received 65 mg Fe with an Indonesian Breakfast, Is Much Better from NaFe(III)EDTA than from Fe(II)SO4, with an Acceptable Increase of Plasma NTBI. A Randomized Clinical Trial
Source: Pharmaceuticals (Basel). 2018 Sep 10;11(3):85. doi: 10.3390/ph11030085 (PMC6161297; doi:10.3390/ph11030085)
Supplement: Supplementary file 1 [file pharmaceuticals-11-00085-s001.zip › suppl/Annex 2; NTBI values.htm]

|  |  |  |  |  |  |  |  |  |  |  |  |  |  |  |  |  |  |  |  |  |  |  |  |  |  |  |  |  |  |  |  |  |  |  |  |
|  | | | | | | | | | | | | | | | | | | | | | | | | | | | | | | | | | | | |
|  |  |  |  |  | No. 1 |  |  | No. 2 |  |  | No. 3 |  |  | No.4 |  |  | No. 5 |  |  | No. 6 |  |  | No. 7 |  |  | No. 8 |  |  | No.9 |  |  | No.10 |  |  | No. 11 |
| Code | Dose Test | Minutes | 1 | 2 | avr | 1 | 2 | avr | 1 | 2 | avr | 1 | 2 | avr | 1 | 2 | avr | 1 | 2 | avr | 1 | 2 | avr | 1 | 2 | avr | 1 | 2 | avr | 1 | 2 | avr | 1 | 2 | avr |
| A-0 | Placebo | 0 | -0,33 | -0,36 | -0,35 | -0,19 | -0,04 | -0,12 | 0,04 | -0,20 | -0,08 | -0,26 | -0,38 | -0,32 | -0,70 | -0,43 | -0,57 | -0,04 | -0,03 | -0,04 | -0,59 | -0,49 | -0,54 | -0,17 | -0,43 | -0,30 | -0,31 | -0,42 | -0,37 | -0,28 | -0,36 | -0,32 | -0,75 | -0,68 | -0,72 |
| A-1 |  | 60 | -0,25 | -0,26 | -0,26 | -0,44 | -0,40 | -0,42 | 0,07 | -0,11 | -0,02 | 0,11 | -0,11 | 0,00 | -0,86 | -0,38 | -0,62 | -0,26 | -0,43 | -0,34 | -0,49 | -0,42 | -0,46 | -0,25 | -0,49 | -0,37 | -0,30 | -0,40 | -0,35 | -0,11 | -0,31 | -0,21 | -0,73 | -0,58 | -0,66 |
| A-2 |  | 120 | -0,46 | -0,40 | -0,43 | 0,12 | 0,10 | 0,11 | 0,15 | 0,04 | 0,10 | -0,37 | -0,33 | -0,35 | -0,88 | -0,50 | -0,69 | -0,21 | -0,32 | -0,26 | -0,48 | -0,54 | -0,51 | -0,10 | -0,08 | -0,09 | -0,28 | -0,52 | -0,40 | -0,06 | -0,15 | -0,11 | -0,61 | -0,57 | -0,59 |
| A-3 |  | 180 | -0,35 | -0,24 | -0,30 | -0,06 | -0,01 | -0,04 | -0,15 | -0,18 | -0,16 | -0,09 | -0,18 | -0,13 | -1,01 | -0,61 | -0,81 | -0,29 | -0,48 | -0,38 | -0,56 | -0,72 | -0,64 | -0,37 | -0,54 | -0,46 | -0,31 | -0,55 | -0,43 | -0,28 | -0,05 | -0,17 | -0,52 | -0,53 | -0,53 |
| A-4 |  | 240 | -0,40 | -0,35 | -0,37 | -0,16 | -0,05 | -0,11 | 0,03 | 0,24 | 0,13 | -0,52 | -0,26 | -0,39 | -1,05 | -0,63 | -0,84 | -0,06 | -0,32 | -0,19 | -0,44 | -0,52 | -0,48 | -0,17 | -0,31 | -0,24 | -0,50 | -0,59 | -0,55 | -0,31 | -0,48 | -0,39 | -0,03 | -0,19 | -0,11 |
| A-5 |  | 300 | -0,31 | -0,27 | -0,29 | -0,06 | -0,09 | -0,07 | 0,05 | 0,36 | 0,20 | -0,16 | -0,31 | -0,24 | -0,93 | -0,57 | -0,75 | -0,20 | -0,36 | -0,28 | -0,55 | -0,58 | -0,57 | -0,27 | -0,66 | -0,46 | -0,38 | -0,52 | -0,45 | -0,09 | -0,18 | -0,13 | -0,26 | -0,18 | -0,22 |
| B-0 | FeSO4 6,5 | 0 | 0,30 | 0,37 | 0,33 | 0,06 | 0,06 | 0,06 | -0,29 | -0,37 | -0,33 | 0,29 | 0,06 | 0,17 | -1,12 | -0,61 | -0,86 | -0,28 | -0,55 | -0,41 | -0,45 | -0,54 | -0,50 | 0,25 | 0,10 | 0,18 | -0,37 | -0,45 | -0,41 | 0,04 | -0,07 | -0,02 | -0,32 | -0,31 | -0,31 |
| B-1 |  | 60 | 0,40 | 0,38 | 0,39 | 0,04 | -0,04 | 0,00 | -0,35 | -0,22 | -0,29 | -0,04 | 0,03 | 0,00 | -0,74 | -0,52 | -0,63 | -0,08 | -0,32 | -0,20 | -0,19 | -0,15 | -0,17 | 0,46 | -0,02 | 0,22 | -0,23 | -0,60 | -0,41 | -0,11 | -0,13 | -0,12 | -0,21 | -0,36 | -0,28 |
| B-2 |  | 120 | 0,23 | 0,09 | 0,16 | 0,16 | 0,26 | 0,21 | -0,61 | -0,41 | -0,51 | -0,26 | -0,30 | -0,28 | -1,16 | -0,60 | -0,88 | -0,23 | -0,43 | -0,33 | -0,52 | -0,52 | -0,52 | 0,68 | 0,22 | 0,45 | no serum |  |  | -0,27 | -0,34 | -0,30 | -0,28 | -0,27 | -0,28 |
| B-3 |  | 180 | -0,38 | -0,39 | -0,39 | 0,11 | 0,29 | 0,20 | -0,57 | -0,43 | -0,50 | 0,07 | -0,18 | -0,06 | -1,19 | -0,39 | -0,79 | -0,22 | -0,51 | -0,37 | -0,58 | -0,67 | -0,63 | 0,63 | 0,27 | 0,45 | -0,33 | -0,38 | -0,36 | -0,18 | -0,44 | -0,31 | -0,57 | -0,48 | -0,52 |
| B-4 |  | 240 | -0,55 | -0,44 | -0,50 | -0,19 | -0,05 | -0,12 | -0,74 | -0,40 | -0,57 | -0,40 | -0,29 | -0,35 | -1,06 | -0,53 | -0,79 | -0,55 | -0,41 | -0,48 | -0,57 | -0,59 | -0,58 | 0,29 | -0,21 | 0,04 | -0,42 | -0,56 | -0,49 | -0,11 | -0,35 | -0,23 | -0,37 | -0,48 | -0,43 |
| B-5 |  | 300 | -0,54 | -0,36 | -0,45 | -0,02 | 0,28 | 0,13 | -0,47 | -0,28 | -0,37 | -0,24 | -0,25 | -0,25 | -1,16 | -0,59 | -0,87 | -0,47 | -0,36 | -0,41 | -0,49 | -0,68 | -0,58 | 0,12 | -0,39 | -0,14 | -0,39 | -0,64 | -0,51 | 0,00 | -0,06 | -0,03 | -0,23 | -0,40 | -0,32 |
| C-0 | FeSO4 65 | 0 | -0,54 | -0,51 | -0,52 | -0,14 | -0,01 | -0,08 | -0,46 | -0,19 | -0,33 | 0,16 | 0,25 | 0,20 | -1,40 | -0,77 | -1,08 | -0,47 | -0,50 | -0,48 | -0,35 | -0,72 | -0,54 | 0,02 | -0,32 | -0,15 | -0,42 | -0,51 | -0,46 | -0,27 | -0,48 | -0,38 | -0,30 | -0,31 | -0,31 |
| C-1 |  | 60 | -0,30 | -0,19 | -0,25 | -0,17 | 0,13 | -0,02 | 0,46 | 1,16 | 0,81 | 0,05 | 0,23 | 0,14 | 0,06 | 0,88 | 0,47 | 0,40 | 0,58 | 0,49 | -0,25 | -0,73 | -0,49 | 0,49 | 0,40 | 0,44 | -0,19 | -0,14 | -0,16 | 1,52 | 1,70 | 1,61 | -0,14 | -0,15 | -0,15 |
| C-2 |  | 120 | -0,07 | 0,19 | 0,06 | 0,64 | 0,81 | 0,72 | 0,15 | 1,12 | 0,64 | 0,48 | 0,82 | 0,65 | 0,61 | 1,53 | 1,07 | 0,02 | 0,24 | 0,13 | 0,07 | -0,42 | -0,17 | 0,12 | 0,21 | 0,17 | -0,37 | -0,41 | -0,39 | 0,82 | 1,01 | 0,91 | 0,13 | -0,02 | 0,05 |
| C-3 |  | 180 | 1,08 | 1,00 | 1,04 | 2,16 | 2,22 | 2,19 | 1,46 | 1,82 | 1,64 | 0,33 | 0,89 | 0,61 | 0,15 | 0,81 | 0,48 | 0,08 | 0,04 | 0,06 | -0,18 | -0,56 | -0,37 | -0,01 | -0,08 | -0,04 | -0,33 | -0,64 | -0,48 | 1,01 | 1,15 | 1,08 | 0,53 | 0,27 | 0,40 |
| C-4 |  | 240 | 0,15 | 0,22 | 0,19 | 2,32 | 2,57 | 2,45 | 1,01 | 2,27 | 1,64 | 0,77 | 1,13 | 0,95 | 0,09 | 0,79 | 0,44 | -0,16 | -0,15 | -0,16 | -0,12 | -0,58 | -0,35 | -0,01 | -0,19 | -0,10 | -0,48 | -0,34 | -0,41 | 0,57 | 0,65 | 0,61 | 0,40 | 0,44 | 0,42 |
| C-5 |  | 300 | 0,36 | 0,29 | 0,32 | 2,59 | 2,75 | 2,67 | 0,61 | 2,09 | 1,35 | 0,31 | 0,56 | 0,44 | -0,53 | 0,35 | -0,09 | -0,08 | -0,29 | -0,19 | -0,17 | -0,59 | -0,38 | -0,11 | -0,25 | -0,18 | -0,34 | -0,46 | -0,40 | 0,63 | 0,50 | 0,56 | 1,35 | 1,45 | 1,40 |
| D-0 | NaFeEDTA 6,5 | 0 | -0,32 | -0,44 | -0,38 | -0,13 | -0,61 | -0,37 | -0,41 | -0,06 | -0,24 | -0,53 | -0,27 | -0,40 | -1,35 | -0,78 | -1,06 | -0,28 | -0,24 | -0,26 | -0,42 | -0,98 | -0,70 | 0,18 | 0,08 | 0,13 | -0,53 | -0,56 | -0,54 | -0,45 | -0,33 | -0,39 | -0,53 | -0,56 | -0,55 |
| D-1 |  | 60 | -0,35 | -0,50 | -0,43 | -0,28 | -0,42 | -0,35 | -0,22 | 0,08 | -0,07 | -0,28 | -0,07 | -0,17 | -1,45 | -0,92 | -1,18 | -0,41 | -0,34 | -0,37 | -0,22 | -0,92 | -0,57 | 0,05 | -0,04 | 0,00 | -0,36 | -0,34 | -0,35 | -0,54 | -0,25 | -0,39 | -0,09 | -0,33 | -0,21 |
| D-2 |  | 120 | -0,35 | -0,52 | -0,44 | -0,23 | -0,47 | -0,35 | -0,06 | -0,14 | -0,10 | -0,59 | -0,09 | -0,34 | -1,32 | -0,77 | -1,04 | -0,54 | -0,40 | -0,47 | -0,30 | -0,79 | -0,55 | 0,03 | -0,12 | -0,05 | -0,33 | -0,26 | -0,30 | -0,57 | -0,51 | -0,54 | -0,32 | -0,16 | -0,24 |
| D-3 |  | 180 | -0,26 | -0,34 | -0,30 | -0,18 | -0,48 | -0,33 | 0,11 | -0,01 | 0,05 | -0,26 | -0,31 | -0,29 | -1,39 | -0,75 | -1,07 | -0,43 | -0,38 | -0,41 | -0,26 | -0,69 | -0,47 | -0,12 | -0,32 | -0,22 | -0,28 | -0,23 | -0,25 | -0,58 | -0,50 | -0,54 | 0,11 | 0,19 | 0,15 |
| D-4 |  | 240 | -0,50 | -0,65 | -0,58 | -0,33 | -0,67 | -0,50 | -0,05 | -0,19 | -0,12 | -0,07 | -0,05 | -0,06 | -1,23 | -0,75 | -0,99 | -0,58 | -0,53 | -0,56 | -0,45 | -1,04 | -0,74 | 0,20 | 0,17 | 0,18 | -0,34 | -0,33 | -0,34 | -0,63 | -0,51 | -0,57 | -0,35 | -0,25 | -0,30 |
| D-5 |  | 300 | -0,38 | -0,46 | -0,42 | -0,40 | -0,57 | -0,49 | 0,27 | 0,12 | 0,19 | -0,25 | 0,23 | -0,01 | -1,19 | -1,10 | -1,15 | -0,56 | -0,66 | -0,61 | -0,40 | -0,87 | -0,63 | 0,17 | 0,19 | 0,18 | -0,50 | -0,57 | -0,54 | -0,68 | -0,49 | -0,59 | -0,28 | -0,38 | -0,33 |
| E-0 | NaFeEDTA 65 | 0 | -0,42 | -0,45 | -0,44 | -0,29 | -0,44 | -0,37 | -0,30 | -0,37 | -0,33 | -0,36 | -0,21 | -0,29 | -0,72 | -0,35 | -0,54 | -0,52 | -0,61 | -0,57 | -0,37 | -0,89 | -0,63 | -0,38 | -0,39 | -0,38 | -0,39 | -0,36 | -0,38 | -0,59 | -0,54 | -0,56 | -0,76 | -0,56 | -0,66 |
| E-1 |  | 60 | -0,26 | -0,37 | -0,31 | 1,27 | 1,65 | 1,46 | 0,30 | 0,29 | 0,29 | 0,00 | 0,19 | 0,09 | -0,18 | 0,04 | -0,07 | -0,43 | -0,61 | -0,52 | -0,13 | -0,68 | -0,40 | -0,14 | -0,21 | -0,17 | -0,28 | -0,35 | -0,31 | 1,41 | 1,60 | 1,51 | 1,03 | 1,26 | 1,14 |
| E-2 |  | 120 | -0,24 | -0,34 | -0,29 | 1,11 | 0,95 | 1,03 | 1,16 | 1,37 | 1,27 | -0,16 | 0,36 | 0,10 | -0,24 | -0,20 | -0,22 | -0,48 | -0,48 | -0,48 | -0,29 | -0,83 | -0,56 | 0,93 | 1,04 | 0,98 | -0,34 | -0,30 | -0,32 | 2,28 | 2,41 | 2,34 | 1,27 | 1,48 | 1,37 |
| E-3 |  | 180 | 0,57 | 0,63 | 0,60 | 1,03 | 1,13 | 1,08 | 0,23 | 0,51 | 0,37 | 0,17 | 0,57 | 0,37 | -0,25 | -0,15 | -0,20 | -0,54 | -0,47 | -0,50 | -0,13 | -0,77 | -0,45 | 1,38 | 1,58 | 1,48 | -0,32 | -0,30 | -0,31 | 1,62 | 1,89 | 1,76 | 1,54 | 1,65 | 1,59 |
| E-4 |  | 240 | 0,87 | 0,93 | 0,90 | 0,50 | 0,70 | 0,60 | 0,71 | 0,56 | 0,64 | 0,22 | 0,79 | 0,50 | -0,36 | -0,40 | -0,38 | -0,49 | -0,62 | -0,56 | -0,16 | -0,38 | -0,27 | 1,30 | 1,57 | 1,44 | -0,41 | -0,36 | -0,38 | 0,75 | 0,97 | 0,86 | 1,56 | 1,73 | 1,64 |
| E-5 |  | 300 | 0,24 | 0,47 | 0,36 | 0,00 | -0,03 | -0,01 | -0,04 | -0,10 | -0,07 | -0,03 | 0,57 | 0,27 | -0,41 | -0,46 | -0,43 | -0,37 | -0,48 | -0,43 | -0,28 | -0,62 | -0,45 | 0,52 | 0,58 | 0,55 | -0,35 | -0,33 | -0,34 | 0,64 | 0,85 | 0,74 | 0,78 | 0,87 | 0,82 |
|  |  |  |  |  |  |  |  |  |  |  |  |  |  |  |  |  |  |  |  |  |  |  |  |  |  |  |  |  |  |  |  |  |  |  |  |
